# Influence of Carbohydrate Additives on the Growth Rate of Microalgae Biomass with an Increased Carbohydrate Content

**DOI:** 10.3390/md19070381

**Published:** 2021-07-01

**Authors:** Anna Andreeva, Ekaterina Budenkova, Olga Babich, Stanislav Sukhikh, Vyacheslav Dolganyuk, Philippe Michaud, Svetlana Ivanova

**Affiliations:** 1 Institute of Living Systems, Immanuel Kant Baltic Federal University, A. Nevskogo Street 14, 236016 Kaliningrad, Russia; AnnaAndreeva@kantiana.ru (A.A.); abudenkova@kantiana.ru (E.B.); olich.43@mail.ru (O.B.); stas-asp@mail.ru (S.S.); dolganuk_vf@mail.ru (V.D.); 2 Department of Bionanotechnology, Kemerovo State University, Krasnaya Street 6, 650043 Kemerovo, Russia; 3 CNRS, SIGMA Clermont, Institut Pascal, Université Clermont Auvergne, F-63000 Clermont-Ferrand, France; 4 Natural Nutraceutical Biotesting Laboratory, Kemerovo State University, Krasnaya Street 6, 650043 Kemerovo, Russia; 5 Department of General Mathematics and Informatics, Kemerovo State University, Krasnaya Street 6, 650043 Kemerovo, Russia

**Keywords:** *Chlorella vulgaris*, *Dunaliella salina*, *Arthrospira platensis*, growth rate, accumulation of carbohydrates, biohydrogen

## Abstract

Our study focused on investigating the possibilities of controlling the accumulation of carbohydrates in certain microalgae species (*Arthrospira platensis* Gomont, *Chlorella vulgaris* Beijer, and Dunaliella *salina* Teod) to determine their potential in biofuel production (biohydrogen). It was found that after the introduction of carbohydrates (0.05 g⋅L^−1^) into the nutrient medium, the growth rate of the microalgae biomass increased, and the accumulation of carbohydrates reached 41.1%, 47.9%, and 31.7% for *Arthrospira platensis, Chlorella vulgaris*, and *Dunaliella salina*, respectively. *Chlorella vulgaris* had the highest total carbohydrate content (a mixture of glucose, fructose, sucrose, and maltose, 16.97%) among the studied microalgae, while for *Arthrospira platensis* and *Dunaliella salina,* the accumulation of total carbohydrates was 9.59% and 8.68%, respectively. Thus, the introduction of carbohydrates into the nutrient medium can stimulate their accumulation in the microalgae biomass, an application of biofuel production (biohydrogen).

## 1. Introduction

Recently, microalgae have attracted attention as a new raw material for biofuel production [1]. Algae biomass has several advantages over land-based energy crops in biofuel production. Microalgae are considered to be the most efficient organisms in converting solar energy. Additionally, microalgae do not require cultivated land, so they do not compete with food crops on arable land [2,3].

Today, microalgae cultivation for lipid production is receiving a large amount of attention [4,5]. After esterification, lipids are used to produce biofuels [6]. Current microalgae research focuses on culture technology to optimize the lipid content of microalgae biomass [7]. However, as an alternative, other algal biomass components can also be used to produce biofuels using biotechnology and thermochemical conversion technology [8,9].

Algae biomass contains different amounts of the most important organic compounds: carbohydrates, proteins, and lipids. Although carbohydrates have a lower energy value compared to other microalgae compounds, they are the best choice or primary raw material for the production of biofuels (such as bioethanol, biobutanol, and biohydrogen) through biotechnological conversion [10,11,12].

Microalgae produce carbohydrates for two main purposes: they serve as a structural component in the cell wall and as a component for intracellular storage [13,14]. As a storage compound, carbohydrates provide the energy needed for the metabolism of the microalgae and allow them to survive temporarily in the dark when needed [15,16]. Microalgae components (such as proteins, lipids, and carbohydrates) allow them to adapt to changing environmental conditions for their growth [17,18]. Carbohydrates are a broad category that includes sugars (monosaccharides) and their polymers (disaccharides, oligosaccharides, and polysaccharides) [19,20].

The content of carbohydrates in biomass depends on the type of microalgae and growth conditions [21,22]. Certain species of microalgae, for example, *Porphyridium cruentum* (40–57%), and *Spirogyra* sp. (33–64%), contain many carbohydrates [23,24]. However, to maximize biofuel production, it is necessary to combine a high carbohydrate content with the ability of microalgae to produce biomass in significant quantities. Therefore, if the microalgae have a high growth rate or other advantages, the cultivation conditions can be controlled to obtain a higher carbohydrate value. Microalgae carbohydrates are used for the production of biofuels, especially biohydrogen [25,26].

The main factors affecting the carbohydrate content of microalgae are nutrient content, salt stress, light intensity, temperature, and metabolism (autotrophic, heterotrophic, and polytrophic). Besides solar energy and carbon dioxide, microalgae also need nutrients, such as nitrogen, phosphorus, and potassium, to grow. The strategy of decreasing or increasing the amount of nutrients is considered an affordable way to produce carbohydrate-rich microalgae [27]. This is possible as it is relatively easy to control the nutrient content of the medium [27,28].

The biomass accumulation level and carbohydrate productivity are important effectiveness indicators of a microalgae strain intended for use in third-generation biofuel production processes. These parameters are influenced by many conditions, including the concentration and composition of nutrients, temperature, pH, and light. A change in these parameters leads to a change in the biomass composition; often the content and composition of various substances (including carbohydrates) can vary significantly in microalgae, which must be considered when scaling the biomass production process. The optimum growth temperature for the most commonly used microalgae is in the 15–35 °C range, depending on the strain. Certain microalgae strains are highly stress-resistant at high temperatures and high concentrations of CO_2_ and NO [29]. 

In general, the pH values for microalgae cultivation are in the range of pH 4.4–7.9, depending on the strain. The pH of the medium influences not only the microalgae, but also the solubility of CO_2_ required for their growth. In certain cases, a steady increase in the microalgae biomass is observed at extremely low pH values. The illumination intensity influences the photosynthesis in chloroplasts of microalgae. The microalgae growth stops with photoinhibition, which occurs after the saturation of their photosystem with photons and the formation of active oxygen forms [30]. In addition to the illumination intensity, the spectral characteristics of light and the light cycle also influence microalgae cultivation. Both a light flux with a wide spectrum (white light) and various LEDs with different spectral characteristics are used, since microalgae absorb light in a narrow range corresponding to the photosynthesis process (up to 700 nm). Several studies have shown that the intensity and spectral characteristics of light affect the accumulation of biomass and carbohydrates [31].

Microalgae absorb organic substances through various mechanisms, such as phosphorylation (for sugars), simple diffusion into cells (for glycerol) or through membrane proteins (for organic acids) [32]. With a mixed [32] or heterotrophic diet [33], Chlorella can be high in carbohydrates [34,35]. However, there is no research into the effect of culture methods on carbohydrate accumulation.

This study aimed to investigate the possibilities of controlling the accumulation of carbohydrates in certain microalgae species (*Arthrospira platensis* Gomont, *Chlorella vulgaris* Beijer, and *Dunaliella salina* Teod) to determine their potential in biofuel production (biohydrogen). To achieve this goal, we planned to sample microalgae, determine their morphology, study the introduction of carbohydrate additives into the nutrient medium, extract chlorophyll, isolate the carbohydrate fraction, determine the carbohydrate content and conduct a statistical analysis of the results.

## 2. Results

### 2.1. Microalgae Identification Results

A comparative analysis of the sequences of 16S rRNA and 18S rRNA genes of microalgae is presented in Appendix A, Appendix B, Appendix C. It was found that *Chlorella vulgaris Beijer, Dunaliella salina Teod,* and *Arthrospira platensis Gomont* were isolated from natural sources (soil, water, and sand).

### 2.2. The Results of the Introduction of Carbohydrates into Nutrient Media for Microalgae Cultivation

**Figure 1:**
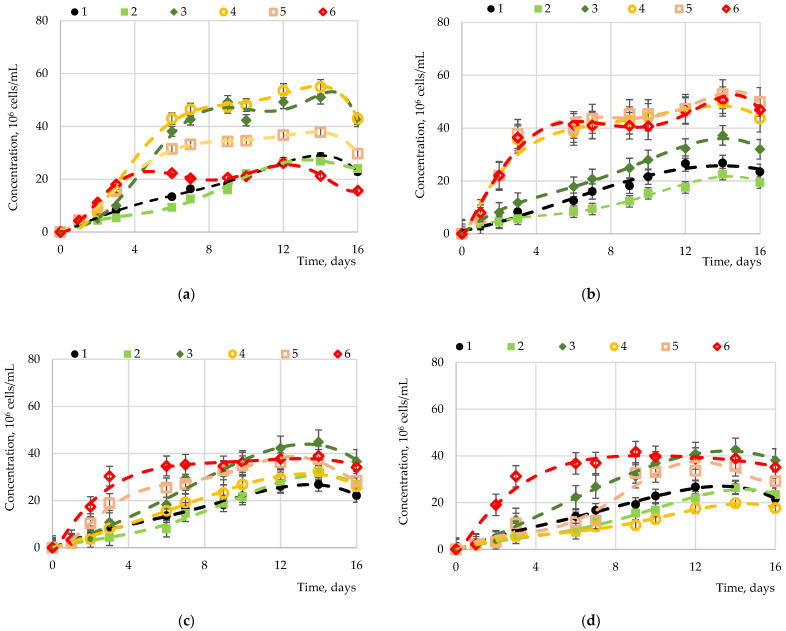
Dependence of the *Chlorella vulgaris* biomass on various sugars: (**a**) fructose, (**b**) glucose, (**c**) maltose, and (**d**) sucrose at various concentrations: 1—control (0.00 g⋅L^−1^); 2—0.05 g·L^−1^; 3—0.10 g·L^−1^; 4—1.00 g·L^−1^; 5—2.00 g·L^−1^; 6—5.00 g·L^−1^.

**Figure 2:**
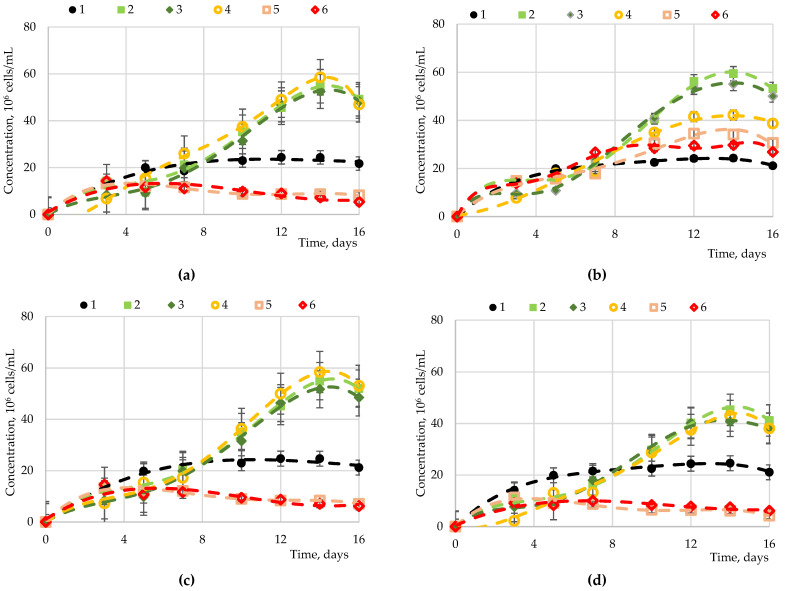
Dependence of the *Arthrospira platensis* biomass on various sugars: (**a**) fructose, (**b**) glucose, (**c**) maltose, and (**d**) sucrose at various concentrations1—control (0.00 g⋅L^−1^); 2—0.05 g·L^−1^; 3—0.10 g·L^−1^; 4—1.00 g·L^−1^; 5—2.00 g·L^−1^; 6—5.00 g·L^−1^.

**Figure 3:**
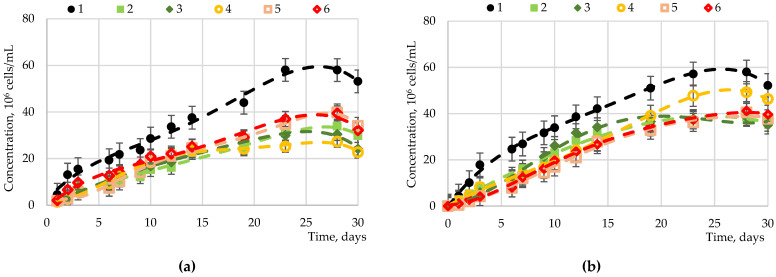

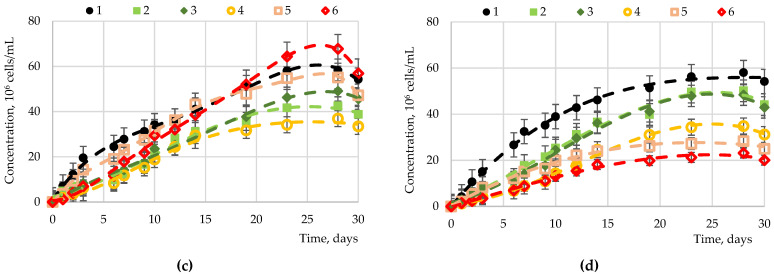
Dependence of the *Dunaliella salina* biomass on various sugars: (**a**) fructose, (**b**) glucose, (**c**) maltose, and (**d**) sucrose at various concentrations: 1—control (0.00 g⋅L^−1^); 2—0.05 g·L^−1^; 3—0.10 g·L^−1^; 4—1.00 g·L^−1^; 5—2.00 g·L^−1^; 6—5.00 g·L^−1^.

**Figure 4:**
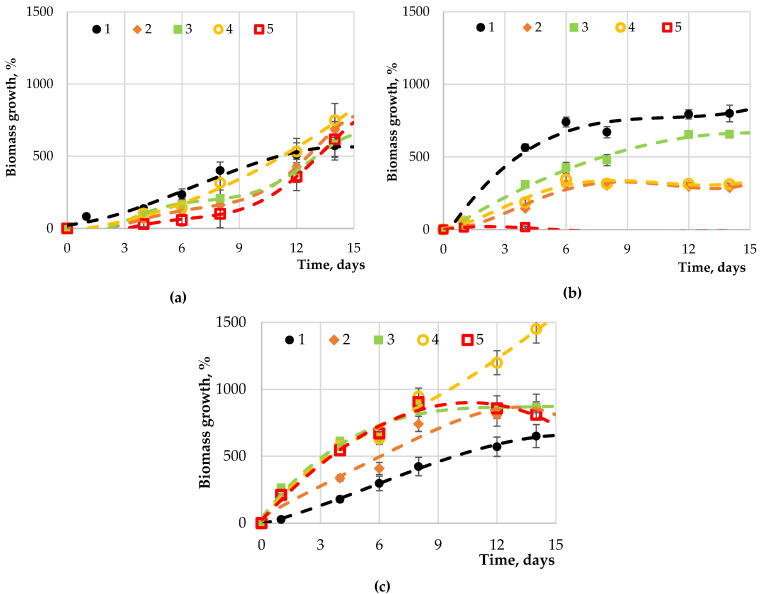
Dependence of the (**a**) *Chlorella vulgaris,* (**b**) *Arthrospira platensis*, and (**c**) *Dunaliella salina* biomass with carbohydrate concentration of 0.05 g·L^−1^ from the concentration medium: 1—control (without carbohydrates); 2—with added fructose; 3—with added glucose; 4—with added maltose; 5—with added sucrose.

Fructose, sucrose, maltose, and glucose were studied as carbohydrates accumulated in microalgae. The results of studies of *Chlorella vulgaris*, *Arthrospira platensis*, and *Dunaliella salina* cultivation processes with the introduction of carbohydrates to the nutrient medium are presented in Figure 1, Figure 2, Figure 3 and Figure 4. 

Statistically significant differences (*p*-value < 0.05, Duncan’s test) in biomass accumulation were observed for *Chlorella vulgaris* (Figure 1) upon application of fructose of different concentrations (from 0 to 5 g·L^−1^). No differences were observed at concentrations of 0.00, 0.05, or 5.00 g·L^−1^, as well as at 0.10 or 1.00 g·L^−1^. The concentrations of 0.00 and 0.05 are not sufficient to significantly affect the biomass growth, while 5.00 g·L^−1^, despite a significant increase at the initial stage, did not lead to significant biomass accumulation, apparently being the concentration of saturation of the medium with this carbohydrate. This is confirmed by the data corresponding to the concentration of 2.00 g·L^−1^. The introduction of glucose at a concentration from 1.00 to 5.00 g·L^−1^ caused significant biomass accumulation, while the biomass growth indicators did not differ significantly. Despite a significant increase in the initial period (up to 3 days) at a concentration of 5.00 g·L^−1^, the concentration of maltose introduced into the cultivation medium did not significantly affect the biomass growth. However, at a concentration of 0.01 g·L^−1^ with a shorter cultivation period (10 days), a slight increase was observed compared to other cultivation modes. The introduction of sucrose in an amount of 5.00 g·L^−1^ led to a significant increase in biomass at the initial stage (3 days), the values of which were not exceeded either at lower concentrations of sucrose or with an increase in the cultivation duration to 14 days. The highest biomass growth rates were achieved with the introduction of fructose and glucose with approximately equal effects.

For *Arthrospira platensis* (Figure 2), statistically significant differences in the accumulation of biomass were observed upon the introduction of fructose at various concentrations (from 0 to 5 g·L^−1^). The introduction of fructose at a concentration of up to 1.0 g·L^−1^ led to an increase in microalgae biomass. At the same time, an increase in the concentration of fructose of 2.00 g·L^−1^ and above led to a significant decrease in biomass accumulation compared to the control. There were no significant differences at concentrations from 0.05 to 1.00 g·L^−1^, or from 2.00 to 5.00 g·L^−1^. The dependence of the biomass accumulation upon the addition of glucose at a concentration from 0.05 to 5.00 g·L^−1^ had the opposite effect; with an increase in the glucose concentration, the biomass accumulated more slowly. The greatest increase in biomass was observed at a concentration of 0.05 and 0.10 g·L^−1^ (*p*-value < 0.05, Duncan’s test). At the initial stage of concentration (up to 3 days), no significant differences in biomass accumulation dependent on the maltose concentration were observed. With further cultivation (more than 7 days), the process of biomass accumulation at a maltose concentration in the cultivation medium from 0.05 to 1.00 g·L^−1^ significantly differed from the control and from the cultivation process at high maltose concentrations (*p*-value = 0.918–0.922 and 0.894–0.942, respectively). The introduction of sucrose in an amount of more than 2.00 g·L^−1^ led to the inhibition of overall biomass growth throughout the entire time interval of cultivation. When cultured for more than 7 days, the biomass accumulation was significantly different (*p*-value > 0.05, Duncan’s test) at a carbohydrate concentration from 0.05 to 2.00 g·L^−1^. The greatest biomass growth indicators were achieved with the introduction of fructose, glucose, or maltose, with approximately equal effects.

The addition of maltose at a concentration of 5.00 g·L^−1^ had a statistically significant effect on *Dunaliella salina* biomass accumulation (Figure 3) compared to the control cultivation. A lower maltose concentration led to a decrease in the growth rate of the biomass of this microalga. The introduction of sucrose led to a significant decrease in the accumulation of biomass in comparison with the control.

The highest indicators of biomass growth were achieved with the introduction of maltose; the rest of the carbohydrates led to a negative impact on the biomass during cultivation.

Thus, for *Chlorella vulgaris*, the maximum biomass growth rate was observed during cultivation with intermittent stirring and the introduction of glucose (1–5 g·L^−1^), fructose (0.1–1.0 g·L^−1^), maltose (2–5 g·L^−1^), and sucrose (5 g·L^−1^). When a higher concentration of carbohydrates (at least 1 g·L^−1^) was introduced into the medium, strong adhesion/aggregation of cells was noted, which complicated mixing and sampling. The contamination risk also increased. The optimal results were achieved by introducing glucose and fructose additives at a concentration of up to 1 g·L^−1^. For *Dunaliella salina*, the maximum biomass growth rate was observed during cultivation under constant stirring and the introduction of maltose to the medium at a rate of 0.05 g·L^−1^ two weeks after the start of growth. The introduction of maltose with a higher concentration and the introduction of fructose, sucrose, and maltose did not significantly affect the growth rate. For *Arthrospira platensis* Gomont, the maximum biomass growth rate was observed during cultivation with constant stirring and the introduction of glucose to the medium at a rate of 0.05–1.00 g·L^−1^ two weeks after the start of growth. The introduction of glucose in a higher concentration and the introduction of fructose, sucrose, and maltose led to the inhibition of culture growth, aggregation of algal cells, and death.

No significant differences (*p*-value > 0.05, Duncan’s test) were observed when introducing carbohydrates (fructose, glucose, maltose, or sucrose) of the same concentration (0.05 g·L^−1^) to the culture medium (Figure 4) of *Chlorella vulgaris.* The introduction of carbohydrates led to a significantly negative effect on the *Arthrospira platensis* biomass growth compared to the control, while maltose had a positive effect on *Dunaliella salina*.

### 2.3. Results of Determining the Content of Carbohydrates in Microalgae 

The results of determining the content of carbohydrates in microalgae are presented in Table 1.

No significant difference (*p*-value < 0.05, Duncan’s test) was found in the accumulation of fructose, maltose, and sucrose in all microalgae under the chosen culture regime. At the same time, the accumulation of glucose significantly differed both between the samples of different microalgae and compared to the control. The same was observed for the complex of carbohydrates; apparently, the greatest contribution of its quantitative formation also relates to glucose.

After the introduction of a carbohydrate additive to the nutrient medium, the accumulation of carbohydrates in the microalgae biomass was 46.4%, 47.9%, 42.3%, and 38.4% for *Chlorella vulgaris*; 35.9%, 41.1%, 37.1%, and 30.2% for *Arthrospira platensis*; 28.6%, 31.7%, 27.6%, and 25.3% for *Dunaliella salina*, respectively, of fructose, glucose, maltose, sucrose, and total carbohydrates (Table 1). *Chlorella vulgaris* was distinguished by the highest content of total carbohydrates (a mixture of glucose, fructose, sucrose, and maltose, 16.97%) among the studied microalgae. While for *Arthrospira platensis* and *Dunaliella salina*, this figure was only 9.59% and 8.68%, respectively. 

*Chlorella vulgaris* had the greatest glucose content (11.26%); the lowest content was observed in *Dunaliella salina* (1.78%). Fructose content prevailed in *Arthrospira platensis* (0.39%); the lowest content was observed in *Chlorella vulgaris* (0.12%). *Chlorella vulgaris* had the greatest sucrose content (0.33%). The highest maltose content was found in *Arthrospira platensis* (0.31%), and the same amount (0.21%) in *Arthrospira platensis* and *Dunaliella salina*. 

The carbohydrate content of microalgae after extraction is shown in Table 2.

The concentration of carbohydrates (Table 1 and Table 2) remained practically unchanged after extraction, which indicates that almost all carbohydrates were transferred to the extract.

The amount of residual carbohydrates in nutrient media after microalgae cultivation was determined spectrophotometrically after acid hydrolysis. The results of determining the residual amount of carbohydrates in the nutrient medium are presented in Table 3. The dynamics of the total consumption of sugars over time by microalgae during cultivation is presented in Table 4.

A significant decrease in the content of residual carbohydrates in nutrient media after microalgae cultivation (Table 3) and the dynamics of total consumption of sugars by microalgae (Table 4) lead to the conclusion that most carbohydrates transfer from the nutrient medium into microalgae.

No significant effect (*p*-value < 0.05, Duncan’s test) of the fructose, maltose or sucrose introduction into the cultivation medium on the carbohydrate content in the culture medium or on the biomass of microalgae (Table 2 and Table 3) was found. Glucose and a mixture of carbohydrates had a statistically significant effect. The residual amount of carbohydrates in nutrient media after growing microalgae decreased by approximately 10 times (Table 2). Such a sharp decrease in the amount of carbohydrates indicates that most of the carbohydrates converted into microalgae biomass during cultivation. After extraction, the amount of carbohydrates was almost identical to that in the algae biomass before extraction.

The total carbohydrate content significantly differed in specialized media (with carbohydrate content) from the control after 8 h of cultivation for all studied algae species. The accumulation of the total amount of carbohydrates during microalgae cultivation period occurred gradually.

## 3. Discussion

Microalgae attract the interest of researchers as a potential source of useful components, including carbohydrates. Ho et al. [36] studied three microalgae isolates for their ability to produce carbohydrates. Among them, *Chlorella vulgaris* FSP-E demonstrated a relatively high rate of cell growth and carbohydrate accumulation. The ability of *C. vulgaris* FSP-E to produce carbohydrates has been further improved through engineering strategies. The results show that using a suitable light intensity and a reasonable inoculum volume was accompanied by cell growth and increased carbohydrate productivity. As a result of nitrogen starvation for 4 days, the carbohydrate content in microalgae reached 51.3%. In our case, the accumulation of carbohydrates ranged from 30.2% in *Arthrospira platensis* to 47.9% in *Chlorella vulgaris*. Under optimal conditions, the maximum productivity of biomass and carbohydrates was 1.437 and 0.631 g·L^−1^ per day^−1^, respectively. Since glucose accounts for almost 93% of the carbohydrates accumulated in *C. vulgaris* FSP-E, microalgae are a promising raw material for bioethanol fermentation.

Markou et al. [18] reported that during the cultivation of microalgae, the carbohydrate content increased with the development of cultivation, and the protein content increased with the later cultivation of diatoms *Rhodomonas sp.*, and decreased in *I. galbana*, *P. lutheri*, and *T. suecica*. *Rhodomonas* sp., and *C. calcitrans* showed lower daily productivity in semi-continuous cultivation. Carbohydrates increased with culture growth, reaching 53.10% and 48.35%, respectively. When comparing the data, we concluded that the results of studying the accumulation of carbohydrates during the cultivation of microalgae, obtained by us and Markou et al. [18], agree, since the studied microalgae and those in our study are multicellular organisms. Microalgae were cultivated at changing operating characteristics (concentration and composition of nutrients, temperature, pH, and light). Changes in these parameters had a significant effect on microalgae biomass composition, as in our studies.

For *Rhodomonas* sp., the highest carbohydrate level peaked in the late resting stage and was 40.24%. In the case of *T. suecica*, carbohydrates accumulate during growth, accounting for 43.23%. A continuous increase in carbohydrate content was observed in the diatoms *P. tricornutum* and *C. calcitruns*, reaching 25.0% and 11.32%, respectively. Except for hybrids, carbohydrate levels (expressed as a percentage) in all tested species increased as the reproductive process intensified. 

Chou et al. [37] confirm this trend for *T. suecicu*. In a semi-continuous culture, the production of all carbohydrates is limited, except for *H. akashiwo*. 

Liu et al. [38] proposed a method for obtaining carbohydrates from *Arthrospira platensis*. In open industrial reservoirs, microalgae grew in conditions of nitrogen deficiency. The maximum yield of biomass and carbohydrates was 27.5 and 26.2 g/m^2^ per day, respectively. By homogenization under pressure, the carbohydrates were extracted with hot water and purified by flocculation. In our study, *Arthrospira platensis* accumulated slightly more carbohydrates—30.2%, as carbohydrates were introduced not independently, but in a complex (glucose, fructose, and maltose).

El-Ahmady El-Naggar et al. [39] extracted and identified water-soluble polysaccharides from the microalga *Chlorella* to use as a plant growth stimulant.

Gaignard et al. [40] studied 166 species of marine microalgae and cyanobacteria to identify strains producing original extracellular polysaccharides. Furthermore, 45 strains with the required characteristics were isolated. Eight new genera of microalgae have been discovered that produce extracellular polysaccharides, including polymers with an original composition.

Dolganyuk et al. [41] found during their study that, in comparison with other cells of microalgae, cultures of microalgae, *Chlorella vulgaris* Beijer, *Arthrospira platensis* Gomont, and *Dunaliella salina* Teod, have characteristics of increased carbohydrate content: 27.36% ± 0.76% and 21.95% ± 0.74%, respectively. The mass fraction of carbohydrates in the biomass of *Cellulopsis obliquus* reached 13.69 ± 0.34 g/m^2^ per day. The content of carbohydrates in the biomass of the microalga *Nannochloropsis gaditana* was 15.34 ± 0.51 g/m^2^ per day. The amount of synthetic carbohydrates in *Chlamydomonas reinhardtii* and *Neochloris cohaerens* was 12.48% ± 0.34% and 12.58% ± 0.34% g/m^2^ per day, respectively [41]. In our study, the microalgae biomass accumulated more carbohydrates (*Chlorella vulgaris* by 13–14%; *Arthrospira platensis* by 3–4%; *Dunaliella salina* by 20–22%), apparently due to the well-chosen dosages of carbohydrates added to the cultivation medium.

In all the studies [36,37,38,39,40,41], stirring during the cultivation of microalgae was a factor that reduces their ability to produce carbohydrates, as in our study.

## 4. Materials and Methods

### 4.1. Microalgae Sampling

To determine the research objects, samples of microalgae were taken from natural sources (water, sand, and soil) in the period from October 2020 to December 2020 in various regions of the Kaliningrad Oblast Lake Vištytis (54°25′37″ N 22°43′30″ E), Lake Chaika (56°03′49″ N 29°04′50″ E), Lake Yantarnoye (56°01′44″ N 30°44′03″ E), Curonian Lagoon (55°07′00″ N 21°01′00″ E), Strait of Baltiysk (59°43′ N 28°24′ E), Baltic Sea coast (54°42.4′0″ N 20° 30.4′0″ E), and Lake Krasnoye (54°25′59″ N 22°30′27″ E)).

Microalgae were sampled with a box-shaped bottom sampler [42], developed at the Institute for Biology of Inland Waters of the Russian Academy of Sciences (IBIW) (Borok, Russia), covering a square area of the bottom 160 × 160 mm in size with a maximum immersion depth of 440 mm in bottom sediments; a 400 mm^2^ sample was taken. Immediately after transportation to the shore, test cores were taken using plastic tubes with an inner diameter of 45 mm. The tubes were sealed at both ends and stored in an upright position at +4 °C. In the laboratory, the core was cut lengthwise and halved using two thin stainless steel plates inserted into the cut. The core halves were then divided into transverse samples (slices) with a step of 5–10 mm. All samples were stored at –20 °C in the dark, in air-tight plastic bags, from which samples of microalgae were taken for research [42].

Pure microalgae cultures were isolated, and strains capable of actively accumulating biomass and target products (lipids, proteins, and carbohydrate–mineral complex) and suitable for cultivation in laboratory conditions from enrichment cultures in which their growth was observed, were identified. The studied samples of natural sources (water, sand, and soil) were introduced into a standard BBM nutrient medium (Stylab, Moscow, Russia) to obtain enrichment cultures to obtain enrichment cultures, purchased from Stylab, Moscow, Russia. For this research, 128 samples of natural sources, taken in various regions of the Kaliningrad region, were used, of which 27 samples showed the growth and development of microalgae at the initial stage of obtaining enrichment cultures.

To identify isolated from the enrichment culture strains of microorganisms (microalgae), partial sequences of the 18S and/or 16S rRNA gene were determined, after which a comparative analysis was performed with the known sequences from the Genbank database. The results of the comparative analysis (Appendix A, Appendix B, Appendix C) of the 18S rRNA gene sequence indicate that the following microalgae were isolated from natural sources (soil, water, and sand): *Chlorella vulgaris* Beijer, *Dunaliella salina* Teod, and *Arthrospira platensis* Gomont.

### 4.2. Microalgae Biomass Cultivation

Microalgae cultivation and the biomass production were carried out on a standard nutrient medium recommended by IPPAS (cellreg.org) (UNIQUE SCIENTIFIC INSTALLATION COLLECTION OF MICROALGAE AND CYANOBACTERIA IPPAS IPP RAS, Moscow, Russia) under conditions of red-white light (~80 ± 10 μE m^−2^ s^−1^), at a temperature of 25 ± 1 °C, with constant and intermittent stirring for 16 days in the case of *Arthrospira platensis* Gomot and *Chlorella vulgaris* Beijer and 30 days in the case of *Dunaliella salina* Teod. Further cultivation is futile and leads to a decrease in the accumulation of microalgae biomass. Shihira-Ishikawa medium (LLC “MicroTech”, Moscow, Russia) was used to culture *Chlorella vulgaris* Beijer; Zarrouk medium (LLC “MicroTech”, Moscow, Russia) was used to culture *Arthrospira platensis* Gomont and produce its biomass; Omarov’s medium (LLC “MicroTech”, Moscow, Russia) was used to produce *Dunaliella salina* Teod biomass. The culture media were sterilized by autoclaving. The microelements of the Zarruk medium were sterilized by filtration through a filter with a pore diameter of 0.22 μm and added after autoclaving into culture media cooled to room temperature [43]. The biomass growth was assessed through the absorption levels of algae samples in the culture medium at 750 nm (Shimadzu, Kyoto, Japan).

Microalgae were cultivated until the required amount of biomass of the studied samples was obtained. 

The cell concentration was counted under an AxioScope A1 microscope (Zeiss, Oberkochen, Germany) applying Goryaev’s cell counting chamber (MiniMed LLC, Bryansk region, Russia) by direct counting of the total number of cells in 1 mL of suspension (OFS.1.7.2.0008.15 determination of concentration microbial cells, Ministry of Health of the Russian Federation). The number of cells in 5 horizontal and 15 diagonal large squares was counted, and the number of cells (X) in 1 mL of the suspension under study was determined by the following equation:

X = a·12499·b,(1)

where a—number of cells in 20 squares; b—dilution of the initial microorganism suspension.

The relative increase in the microalgae biomass was determined by the following equation [44]:

(2) $$X=\frac{\left(m1-m2\right)}{m1}\cdot 100\%,$$

where m1—microalgae mass through the entire growing period, mg; m2—microalgae mass each cultivation day.

### 4.3. Microalgae Morphology Determination

The morphology of microalgae was determined at 40× magnification using a binocular microscope MC-300 (Micros, Vienna, Austria) [45]. 

### 4.4. Introduction of Carbohydrate Additives to the Nutrient Medium

Fructose, sucrose, maltose, and glucose (LLC “Moskhimtorg”, Moscow, Russia) were used as carbohydrate additives. Carbohydrates were added to the nutrient medium in amounts of 0.05, 0.10, 1.00, 2.00, and 5.00 g·L^−1^. Control—cultivation medium without added carbohydrates.

### 4.5. Chlorophyll Extraction

A 1 mL microalgae suspension sample was centrifuged for 20 min at 3400 rpm, and the supernatant was removed. After that, 10 mL of ethanol (96%) was added. The mixture was incubated for 10 min at 25 °C in a water bath and centrifuged for 20 min at 3400 rpm (the sediment is the discolored algae biomass; the liquid phase is the pigment extract). Then, 0.3 g of activated carbon was added to the liquid phase, gently mixed for several seconds, and filtered (to prevent partial ingress of pigments in the liquid phase into the precipitate) [46]. All reagents were purchased from LLC “Moskhimtorg”, Moscow, Russia.

### 4.6. Carbohydrate Fraction Isolation

The carbohydrate fraction was isolated as follows. An amount of 1 mL of sulfuric acid (72%) was added to a weighed portion of dry algae (100 mg) in a glass flask, incubated at 30 °C for 1 h, and 28 mL of distilled water was added, then it was autoclaved for 1 h at 120 °C. Furthermore, it was quickly cooled, and 1 mL of the sample was taken and centrifuged at 8000 rpm for 5 min. Hydrochloric acid was used to isolate fructose. All reagents were purchased from LLC “Moskhimtorg”, Moscow, Russia. Additionally, the method of high-performance liquid chromatography (HPLC) was used applying an HPLC NGC chromatograph (Bio-rad, Berkeley, CA, USA) on a Uno-Q1 column (Bio-rad, Berkeley, CA, USA) in the mode of gradient pH 2.5–8.9. The eluent comprised the following buffer solutions: phase A—citrate-phosphate buffer with pH 2.5; B—tris-glycine buffer pH 8.5; gradient phase B 0—100% for 15 column volumes (1 column volume—1 mL). Chromatography parameters were as follows: run: 04; trace type: λ 3 (280 nm); best fit: 8; slope: 10; sensitivity: medium; size: N/A.

### 4.7. Determination of Carbohydrate Content 

Experimental samples were cultured under conditions similar to those previously described. The carbohydrate additive was a mixture of glucose, fructose, and maltose; the concentration of each carbohydrate was 0.1 g·L^−1^. The concentrated carbohydrate solution was sterilized and added to the experimental flasks with the medium (20 μL each). Samples for analysis were taken at regular intervals once a day under sterile conditions.

The content of carbohydrates in the sample was determined by the phenol-sulfuric acid method; a weighed portion of dry algae (10 mg) was dissolved in distilled water (10 mL). Then, 1 mL of the sample was introduced into a glass flask, and 3 mL of sulfuric acid (72%) and 1 mL of phenol (5%) were added. To determine the fructose content, 1 mL of resorcinol (0.1%) and 3 mL of concentrated hydrochloric acid were added to the sample and kept in a water bath for 5 min at 90 °C. Next, calibration solutions were prepared with a known concentration of carbohydrates (glucose, fructose, sucrose, and maltose mixture), and the absorption was measured on a spectrophotometer at 490 nm relative to glucose and fructose, and at 440 nm for sucrose and at 545 nm for maltose (the absorption maximum was checked using standard solutions in fivefold repetition). The carbohydrate content determination was carried out on cultures in the exponential growth phase (determined spectrophotometrically, 750 nm). The spectrophotometer was calibrated using the dry weight method. The dry residue of the biomass of multicellular microalgae was dissolved in carbon tetrachloride (to obtain a solution with a microalgae content of 200 mg/mL), and the light absorption of the solution was investigated at 5.0–9.0 nm with respect to carbon tetrachloride. The applied method guaranteed the accuracy of the data obtained [43]. All reagents were purchased from LLC “Moskhimtorg”, Moscow, Russia. 

### 4.8. Determination of the Residual Amount of Sugars in Nutrient Media

Acid hydrolysis was used to extract residual sugar from the culture medium. A fixed amount of the collected culture medium (15 g/L, resuspended in distilled H_2_O) was used as a substrate for analyses. Various concentrations of sulfuric acid (47, 94, 188, 281, and 563 mM) were tested to determine the most effective concentration. Hydrolysis analyses were performed in Erlenmeyer flasks. The reaction proceeded at 100 °C for 30 min (Waiser Lab. Products NC EST–011). Samples were cooled at room temperature and then centrifuged at 3200× *g* at 20 °C for 8 min (Excelsa ®II model 206 BL). The supernatant containing residual sugars was collected, and its pH was adjusted to 5.5 using 1 M NaOH. The residual sugar concentration was analyzed by the DNS (dinitrosalicylic acid) method with glucose as the standard for the calibration curves. After mixing 0.75 mL of glucose with 0.5 mL of DNS reagent, the samples were heated at 100 °C for 5 min. The samples were cooled to room temperature, and then 3 mL of water was added. Sugar concentrations were determined spectrophotometrically (Varian, Inc. Cary ® 50 UV-Vis) at 540 nm.

### 4.9. Statistical Analysis

One-way analysis of variance (ANOVA) was performed to minimize the risk of misjudgment of a type 1 error in the case of multiple comparisons. The correspondence of the samples used to the normal distribution was checked by the *t*-test (mathematical expectations) for independent samples and Fisher’s test (variance). Post hoc analysis (Duncan’s test) was undertaken to identify samples that were significantly different from each other. The equality of the variances of the extracted samples was checked using the Levene test [47]. Significant differences were considered as a *p*-value < 0.05.

## 5. Conclusions

The accumulation of carbohydrates in certain species of microalgae (*Arthrospira platensis* Gomont, *Chlorella vulgaris* Beijer, and *Dunaliella salina* Teod) was studied to determine their potential for biofuel production (biohydrogen). It was found that after the introduction of carbohydrates (0.05 g·L^−1^) into the nutrient medium, the growth rate of the microalgae biomass increased, and the accumulation of carbohydrates reached 41.1%, 47.9%, and 31.7% for *Arthrospira platensis, Chlorella vulgaris*, and *Dunaliella salina*, respectively. *Chlorella vulgaris* had the highest total carbohydrate content (the sum of glucose, fructose, sucrose, and maltose was 16.97%).

The introduction of carbohydrates to culture media can be used to produce microalgal biomass enriched with these biopolymers. For certain technologies, carbohydrates play a key role in biomass conversion. 

However, the accumulation of carbohydrates in biomass is significantly influenced by the species of microalgae and the initial growth conditions; therefore, further research focused on optimizing, and, where possible, standardizing the conditions for cultivating microalgae is needed. The high carbohydrate content in microalgae, especially monosaccharides, such as glucose, contributes to the biofuel production process. Carbohydrate-rich microalgae can also be used as raw materials to produce ethylene glycol and 1,2-propanediol using environmentally friendly chemical reactions. Therefore, an urgent and important task is to obtain the biomass of microalgae enriched with carbohydrates or starch, which can be used as a raw material for subsequent chemical or biochemical transformations [13].

## Figures and Tables

**Table 1:** Microalgae carbohydrate content before extraction.

Carbohydrates	Concentration, % of Dry Matter					
	I		II		III	
	exp.	Control	exp.	Control	exp.	Control
1	0.12 ± 0.02 ^a^	0.10 ± 0.01	0.39 ± 0.08 ^a^	0.42 ± 0.05	0.38 ± 0.01 ^a^	0.33 ± 0.01
2	11.26 ± 0.72 ^b^	9.78 ± 0.22	3.41 ± 0.62 ^b^	2.32 ± 0.30	1.78 ± 0.29 ^b^	1.60 ± 0.22
3	0.12 ± 0.03 ^a^	0.11 ± 0.02	0.21 ± 0.02 ^a^	0.20 ± 0.02	0.21 ± 0.01 ^a^	0.22 ± 0.01
4	0.33 ± 0.06 ^a^	0.27 ± 0.03	0.31 ± 0.09 ^a^	0.32 ± 0.07	0.11 ± 0.06 ^a^	0.10 ± 0.02
5	16.97 ± 0.64 ^c^	15.12 ± 0.48	9.59 ± 0.20 ^c^	7.81 ± 0.76	8.68 ± 0.74 ^c^	7.54 ± 0.30

1—fructose; 2—lucose; 3—maltose; 4—sucrose; 5—total glucose, fructose, sucrose, and maltose; I—*Chlorella vulgaris*; II—*Arthrospira platensis*; III—*Dunaliella salina*; exp.—experimental samples; control—no added carbohydrates. Data presented as a mean ± SD (n = 3). Values in a columns followed by the same letter do not differ significantly *(p*-value > 0.05), as assessed by the post hoc test (Duncan’s test).

**Table 2:** Amount of carbohydrate in microalgae after extraction.

Carbohydrates	Concentration, % of Dry Matter					
	I		II		III	
	exp.	Control	exp.	Control	exp.	Control
1	0.11 ± 0.02 ^a^	0.10 ± 0.01	0.37 ± 0.08 ^a^	0.42 ± 0.05	0.33 ± 0.01 ^a^	0.33 ± 0.01
2	10.48 ± 0.72 ^b^	9.78 ± 0.22	3.40 ± 0.62 ^b^	2.32 ± 0.30	1.76 ± 0.29 ^b^	1.60 ± 0.22
3	0.11 ± 0.03 ^a^	0.11 ± 0.02	0.19 ± 0.02 ^a^	0.20 ± 0.02	0.20 ± 0.01 ^a^	0.22 ± 0.01
4	0.29 ± 0.06 ^a^	0.27 ± 0.03	0.30 ± 0.09 ^a^	0.32 ± 0.07	0.10 ± 0.06 ^a^	0.10 ± 0.02
5	15.99 ± 0.64 ^c^	15.12 ± 0.48	8.98 ± 0.20 ^c^	7.81 ± 0.76	8.66 ± 0.74 ^c^	7.54 ± 0.30

1—fructose; 2—lucose; 3—maltose; 4—sucrose; 5—total glucose, fructose, sucrose, and maltose; I—*Chlorella vulgaris*; II—*Arthrospira platensis*; III—*Dunaliella salina*; exp.—experimental samples; control—no added carbohydrates. Data presented as a mean ± SD (n = 3). Values in a columns followed by the same letter do not differ significantly (*p* > 0.05), as assessed by the post hoc test (Duncan’s test).

**Table 3:** Residual amounts of carbohydrates in nutrient media.

Carbohydrates	Concentration, % of Dry Matter		
	I	II	III
1	1.0 ± 0.2 ^a^	3.0 ± 0.6 ^a^	2.3 ± 0.3 ^a^
2	1.3 ± 0.2 ^a^	4.1 ± 0.5 ^b^	9.1 ± 0.6 ^b^
3	2.1 ± 0.3 ^b^	1.2 ± 0.2 ^c^	2.0 ± 0.1 ^a^
4	2.0 ± 0.3 ^b^	2.0 ± 0.3 ^c^	1.1 ± 0.2 ^c^
5	1.9 ± 0.4 ^b^	1.1 ± 0.2 ^c^	0.9 ± 0.4 ^c^

1—fructose; 2—glucose; 3—maltose; 4—sucrose; 5—total glucose, fructose, sucrose, and maltose; I—*Chlorella vulgaris* culture medium; II—*Arthrospira platensis* culture medium; III—*Dunaliella salina* culture medium; exp. —experimental samples; control—no added carbohydrates. Data presented as a mean ± SD (n = 3). Values in a columns followed by the same letter a, b or c do not differ significantly (*p*-value > 0.05), as assessed by the post hoc test (Duncan’s test).

**Table 4:** Total consumption of sugars by microalgae over time.

Cultivation Time, Days	Concentration, % of Dry Matter		
	I	II	III
0	0.08 ± 0.02 ^a^	0.09 ± 0.03 ^a^	0.02 ±0.06 ^a^
4	2.43 ± 0.31 ^b^	1.59 ± 0.05 ^b^	0.45 ± 0.14 ^b^
8	9.88 ± 0.46 ^c^	5.29 ± 0.18 ^c^	0.78 ± 0.23 ^b^
12	16.97 ± 0.64 ^d^	9.59 ± 0.20 ^d^	1.13 ± 0.51 ^c^
16	4.58 ± 0.34 ^e^	2.62 ± 0.12 ^e^	1.27 ± 0.62 ^c^
20	-	-	1.69 ± 0.66 ^c^
24	-	-	2.37 ±0.71 ^d^
28	-	-	8.68 ± 0.74 ^e^
30	-	-	0.97 ± 0.47 ^b^

I—*Chlorella vulgaris*; II—*Arthrospira platensis*; III—*Dunaliella salina*; exp.—experimental samples; control—no added carbohydrates. Data presented as a mean ± SD (n = 3). Values in a columns followed by the same letter a, b, c, d or e do not differ significantly (*p*-value > 0.05), as assessed by the post hoc test (Duncan’s test).

## Data Availability

The data are included in the manuscript.

## Appendix A. Sequences of the 18S Ribosomal RNA Gene of the *Chlorella vulgaris* Beijer

TGTAGTCATATGCTTGTCTCAAAGATTAAGCCATGCATGTCTAAGTATAAACTGCTTTATACTGTGAAACTGCGAATGGCTCATTAAATCAGTTATAGTTTATTTGATGGTACCTACTACTCGGATACCCGTAGTAAATCTAGAGCTAATACGTGCGTAAATCCCGACTTCTGGAAGGGACGTATTTATTAGATAAAAGGCCGACCGGGCTCTGCCCGACTCGCGGTGAATCATGATAACTTCACGAATCGCATGGCCTTGTGCCGGCGATGTTTCATTCAAATTTCTGCCCTATCAACTTTCGATGGTAGGATAGAGGCCTACCATGGTGGTAACGGGTGACGGAGGATTAGGGTTCGATTCCGGAGAGGGAGCCTGAGAAACGGCTACCACATCCAAGGAAGGCAGCAGGCGCGCAAATTACCCAATCCTGACACAGGGAGGTAGTGACAATAAATAACAATACTGGGCCTTTTCAGGTCTGGTAATTGGAATGAGTACAATCTAAACCCCTTAACGAGGATCAATTGGAGGGCAAGTCTGGTGCCAGCAGCCGCGGTAATTCCAGCTCCAATAGCGTATATTTAAGTTGCTGCAGTTAAAAAGCTCGTAGTTGGATTTCGGGTGGGGCCTGCCGGTCCGCCGTTTCGGTGTGCACTGGCAGGGCCCACCTTGTTGCCGGGGACGGGCTCCTGGGCTTCACTGTCCGGGACTCGGAGTCGGCGCTGTTACTTTGAGTAAATTAGAGTGTTCAAAGCAGGCCTACGCTCTGAATACATTAGCATGGAATAACACGATAGGACTCTGGCCTATCCTGTTGGTCTGTAGGACCGGAGTAATGATTAAGAGGGACAGTCGGGGGCATTCGTATTTCATTGTCAGAGGTGAAATTCTTGGATTTATGAAAGACGAACTACTGCGAAAGCATTTGCCAAGGATGTTTTCATTAATCAAGAACGAAAGTTGGGGGCTCGAAGACGATTAGATACCGTCCTAGTCTCAACCATAAACGATGCCGACTAGGGATCGGCGGATGTTTCTTCGATGACTCCGCCGGCACCTTATGAGAAATCAAAGTTTTTGGGTTCCGGGGGGAGTATGGTCGCAAGGCTGAAACTTAAAGGAATTGACGGAAGGGCACCACCAGGCGTGGAGCCTGCGGCTTAATTTGACTCAACACGGGAAAACTTACCAGGTCCAGACATAGTGAGGATTGACAGATTGAGAGCTCTTTCTTGATTCTATGGGTGGTGGTGCATGGCCGTTCTTAGTTGGTGGGTTGCCTTGTCAGGTTGATTCCGGTAACGAACGAGACCTCAGCCTGCTAAATAGTCACGGTTGGCTCGCCAGCCGGCGGACTTCTTAGAGGGACTATTGGCGACTAGCCAATGAAGCATGAGGCAATAACAGGTCTGTGATGCCCTTAGATGTTCTGGGCCGCACGCGCGCTACACTGATGCATTCAACGAGCTTAGCCTTGGCCGAGAGGCCCGGGTAATCTTTGAAACTGCATCGTGATGGGGATAGATTATTGCAATTATTAATCTTCAACGAGGAATGCCTAGTAAGCGCAAGTCATCAGCTTGCGTTGATTACGTCCCTGCCCTTTGTACACACCGCCCGTCGCTCCTACCGATTGGGTGTGCTGGTGAAGTGTTCGGATTGGCGACCGGGGGCGGTCTCCGCTCTCGGCCGCCGAGAAGTTCATTAAACCCTCCCACCTAGAGGAAGGAGAAGTCGTAACAAGGTTTCCGTAGGTGAACCTGCGGAAGGATCA

## Appendix B. Sequences of the 18S Ribosomal RNA Gene of the *Arthrospira platensis* Gomont

AGAGTTTGATCCTGGCTCAGGATGAACGCTGGCGGTCTGCTTAACACATGCAAGTCGAACGGGCTCTTCGGAGCTAGTGGCGGACGGGTGAGTAACACGTGAGAATCTGGCTCCCGGTCGGGGACAACAGAGGGAAACTTCTGCTAATCCCGGATGAGCCGAAAGGTAAAAGATTTATCGCCGGGAGATGAGCTCGCGTCTGATTAGCTAGTTGGTGAGGTAAAGGCTCACCAAGGCGACGATCAGTAGCTGGTCTGAGAGGATGATCAGCCACACTGGGACTGAGACACGGCCCAGACTCCTACGGGAGGCAGCAGTGGAGAATTTTCCGCAATGGGCGCAAGCCTGACGGAGCAAGACCGCGTGGGGGAGGAAGGCTCTTGGGTTGTAAACCCCTTTTCTCAAGGAAGAACACAATGACGGTACTTGAGGAATAAGCCTCGGCTAACTCCGTGCCAGCAGCCGCGGTAATACGGAGGAGGCAAGCGTTATCCGGAATGATTGGGCGTAAAGCGTCCGTAGGTGGCAGTTCAAGTCTGCTGTCAAAGACAGTAGCTCAACTACTGAAAGGCAGTGGAAACTGAACAGCTAGAGTACGGTAGGGGCAGAGGGAATTCCCGGTGTAGCGGTGAAATGCGTAGATATCGGGAAGAACACCGGTGGCGAAAGCGCTCTGCTGGGCCGTAACTGACACTGAGGGACGAAAGCTAGGGGAGCGAATGGGATTAGATACCCCAGTAGTCCTAGCCGTAAACGATGGAAACTAGGTGTAGCCTGTATCGACCCGGGCTGTGCCGAAGCTAACGCGTTAAGTTTCCCGCCTGGGGAGTACGCACGCAAGTGTGAAACTCAAAGGAATTGACGGGGGCCCGCACAAGCGGTGGAGTATGTGGTTTAATTCGATGCAACGCGAAGAACCTTACCAGGGCTTGACATGTCCGGAATCTTGGTGAAAGCCGAGAGTGCCTTCGGGAGCCGGAACACAGGTGGTGCATGGCTGTCGTCAGCTCGTGTCGTGAGGTGTTGGGTTAAGTCCCGCAACGAGCGCAACCCTCGTCCTTAGTTGCCATCATTCAGTTGGGCACTTTAGGGAGACTGCCGGTGACAAACCGGAGGAAGGTGGGGATGACGTCAAGTCATCATGCCCCTTACGTCCTGGGCTACACACGTACTACAATGGGGGGGACAAAGGGTAGCCAAGACGCGAGTCTGAGCCAATCCCGTAAACCTCTCCTCAGTTCAGATTGCAGGCTGCAACTCGCCTGCATGAAGGAGGAATCGCTAGTAATCGCAGGTCAGCATACTGCGGTGAATCCGTTCCCGGGCCTTGTACACACCGCCCGTCACACCATGGAAGTTAGCCACGCCCGAAGTCGTTACTCTAACCGTTCGCGGAGGAGGATGCCGAAGGCAGGGCTGATGACTGGGGTGAAGTCGTAACAAGGTAGCCGTACCGGAAGGTGTGGCTGGATCACCTCCTTTTTAGGGAGACCTACTTCGAGATATCGCGCCTTAACAACTATAGCCGTGTCTTGAGGTCATCCTTAGGTCGGATGGGGCGGTCAGAGAGCTTTCAAACTTTAGGGTTCGTGTTATGGGCTATTAGCTCAGGTGGTTAGAGCGCACCCCTGATAAGGGTGAGGTCCCTGGTTCAAGTCCAGGATGGCCCACATCCACCCCAAACTGGGGGTATAGCTCAGTTGGTAGAGCGCTGCCTTTGCACGGCAGAAGTCAGCGGTTCGAGTCCGCTTACCTCCACTCTCCTTTGTGATGGTGCTAGTTGGGGTGAGATGAGATGAGATGACCTCTGATAGATAATTTATCACTGTACAGCTCCTAAATCTTTAGATGTTAGTCTGAGATTGGATAGCTGGACATCTGTTCCAGTCAGAACCTTGAAAACTGCATAGAGAAAAGCATAATGGTGTAGGAAAACGTCGTAAAGACAATTCCAATGTAGGTCAAGCTACAAAGGGCTAACGGTGGAACCTAGGCACACAGAGCGGCCGCAAA

## Appendix C. Sequences of the 18S Ribosomal RNA Gene of the *Dunaliella salina* Teod

CTGGTTGATCCTGCCAGTAGTCATATGCTTGTCTCAAAGATTAAGCCATGCATGTCTAAGTATAAACTGCTTATACTGTGAAACTGCGAATGGCTCATTAAATCAGTTATAGTTTATTTGATGGTACCTTTACTCGGATAACCGTAGTAATTCTAGAGCTAATACGTGCGTAAATCCAGACTTCTGGAAGGGACGTATTTATTAGATAAAAGGCCAGCCGGGCTTGCCCGACTCTTGGCGAATCATGATAACTTCACGAATCGCACGGCTTTATGCCGGCGATGTTTCATTCAAATTTCTGCCCTATCAACTTTCGATGGTAGGATAGAGGCCTACCATGGTGGTAACGGGTGACGGAGGATTAGGGTTCGATTCCGGAGAGGGAGCCTGAGAAACGGCTACCACATCCAAGGAAGGCAGCAGGCGCGCAAATTACCCAATCCCAACACGGGGAGGTAGTGACAATAAATAACAATACCGGGCATTTTTGTCTGGTAATTGGAATGAGTACAATCTAAATCCCTTAACGAGTATCCATTGGAGGGCAAGTCTGGTGCCAGCAGCCGCGGTAATTCCAGCTCCAATAGCGTATATTTAAGTTGTTGCAGTTAAAAAGCTCGTAGTTGGATTTCGGGTGGGTTGTAGCGGTCAGCCTTTGGTTAGTACTGCTACGGCCTACCTTTCTGCCGGGGACGAGCTCCTGGGCTTAACTGTCCGGGACTCGGAATCGGCGAGGTTACTTTGAGTAAATTAGAGTGTTCAAAGCAAGCCTACGCTCTGAATACATTAGCATGGAATAACACGATAGGACTCTGGCTTATCTTGTTGGTCTGTAAGACCGGAGTAATGATTAAGAGGGACAGTCGGGGGCATTCGTATTTCATTGTCAGAGGTGAAATTCTTGGATTTATGAAAGACGAACTTCTGCGAAAGCATTTGCCAAGGATGTTTTCATTAATCAAGAACGAAAGTTGGGGGCTCGAAGACGATTAGATACCGTCGTAGTCTCAACCATAAACGATGCCGACTAGGGATTGCCAGGTGTTTCGTTGATGACCCTGCCAGCACCTTATGAGAAATCAAAGTTTTTGGGTTCCGGGGGGAGTATGGTCGCAAGGCTGAAACTTAAAGGAATTGACGGAAGGGCACCACCAGGCGTTAACTTAGCAGCAAGCTCAGCGCCTCAAAGTCGAAGGGAAACCTTTGGCTAGTATCTGGGTGTAGATTTCACCTAAGTGCAACACTGTTCAAATTGCGGGAAAGCCCTAAAGCTTTGCTAACCAAGCTGTCCTAGAAATGGGATGGTGGCCAGGTGAAAGACCTTGGGTACGGTAAAATCAGCAAAGATGCAACAATGGGCAATCCGCAGCCAAGCTCCTACGGGCTGTCAAAGCCTATGGAGAAGGTTCAGAGACTAAATGGCAGTGGGCAAGCATGGCAATGCTTGCTTAAGATATAGTCCGTCCCAGCTGAGAAGCTGCCTATGAGAGGAATGCCGTAAGGCAGGAGAGCTAATAGGAAGTAAGTGTCTTTAATCAACTTACTTGGATTCCACGGGAGCCTGCGGCTTAATTTGACTCAACACGGGAAAACTTACCAGGTCCAGACACGGGGAGGATTGACAGATTGAGAGCTCTTTCTTGATTCTGTGGGTGGTGGTGCATGGCCGTTCTTAGTTGGTGGGTTGCCTTGTCAGGTTGATTCCGGTAACGAACGAGACCTCAGCCTGCTAAATAGTCACGTCTACCTCGGTAGGCGCCTGACTTCTTAGAGGGACTATTGGCGTTTAGCCAATGGAAGTGTGAGGCAATAACAGGTCTGTGATGCCCTTAGATGTTCTGGGCCGCACGCGCGCTACACTGATGCATTCAACGAGCCTATCCTTGGCCGAGAGGTCCGGGTAATCTTTGAAACTGCATCGTGATGGGGATAGATTATTGCAATTATTAGTCTTCAACGAGGAATGCCTAGTAAGCGCGAGTCATCAGCTCGCGTTGATTACGTCCCTGCCCTTTGTACACACCGCCCGTCGCTCCTACCGATTGGGTGTGCTGGTGAAGTGTTTGGATCGGTACCAATGGGGGGAAACCTCTGTTGGTACTGAGAAGAACATTAAACCCTCCCACCTAGAGGAAGGAGAAGTCGTAACAAGGTTTCCGTAGGTGAACCT GCAGAAGGATCA

## References

[1] Zhou X., Lin W., Tong L., Liu X., Zhong K., Liu L., Wang L., Zhou S. (2016). Hypolipidaemic effects of oat flakes and beta-glucans derived from four Chinese naked oat (*Avena nuda*) cultivars in Wistar-Lewis rats. J. Sci. Food Agric..

[2] Kharwar S., Bhattacharjee S., Mishra A.K. (2021). disentangling the impact of sulfur limitation on exopolysaccharide and functionality of Alr2882 by in silico approaches in *Anabaena* sp. PCC 7120. Appl. Biochem. Biotechnol..

[3] Vieira M.V., Pastrana L.M., Fuciños P. (2020). Microalgae encapsulation systems for food, pharmaceutical and cosmetics applications. Mar. Drugs.

[4] Ma R., Wang B., Chua E.T., Zhao X., Lu K., Ho S.H., Shi X., Liu L., Xie Y., Lu Y. (2020). Comprehensive utilization of marine microalgae for enhanced co-production of multiple compounds. Mar. Drugs.

[5] Rumin J., Nicolau E., Junior R.G.O., Fuentes-Grünewald C., Picot L. (2020). Analysis of scientific research driving microalgae market opportunities in Europe. Mar. Drugs.

[6] Bodachivskyi I., Kuzhiumparambil U., Bradley G., Williams D. (2019). High yielding acid-catalysed hydrolysis of cellulosic polysaccharides and native biomass into low molecular weight sugars in mixed ionic liquid systems. Chem. Open.

[7] Koyande A.K., Show P.L., Guo R., Tang B., Ogino C., Chang J.S. (2019). Bio-processing of algal bio-refinery: A review on current advances and future perspectives. Bioengineered.

[8] Lam G.P., Giraldo J.B., Vermue M.H., Olivieri G., Eppink M.H., Wijffels R.H. (2016). Understanding the salinity effect on cationic polymers in inducing flocculation of the microalga *Neochloris oleoabundans*. J. Biotechnol..

[9] Lakatos G.E., Ranglová K., Manoel J.C., Grivalský T., Kopecký J., Masojídek J. (2019). Bioethanol production from microalgae polysaccharides. Folia. Microbiol..

[10] Andreeva A., Budenkova E., Babich O., Sukhikh S., Ulrikh E., Ivanova S., Prosekov A., Dolganyuk V. (2021). Production, purification, and study of the amino acid composition of microalgae proteins. Molecules.

[11] Klanchui A., Dulsawat S., Chaloemngam K., Cheevadhanarak S., Prommeenate P., Meechai A. (2018). An improved genome-scale metabolic model of *Arthrospira platensis* C1 (iAK888) and its application in glycogen overproduction. Metabolites.

[12] Aytenfisu A.H., Yang M., MacKerell A.D. (2018). Drude polarizable force field for glycosidic linkages involving pyranoses and furanoses. J. Chem. Theory Comput..

[13] Cheng D., Li D., Yuan Y., Zhou L., Li X., Wu T., Wang L., Zhao Q., Wei W., Sun Y. (2017). Improving carbohydrate and starch accumulation in *Chlorella* sp. AE10 by a novel two-stage process with cell dilution. Biotechnol. Biofuels.

[14] Schulze C., Wetzel M., Reinhardt J., Schmidt M., Felten L., Mundt S. (2016). Screening of microalgae for primary metabolites including β-glucans and the influence of nitrate starvation and irradiance on β-glucan production. J. Appl. Phycol..

[15] Khan M.I., Lee M.G., Seo H.J., Shin J.H., Shin T.S., Yoon Y.H., Kim M.Y., Choi J.I., Kim J.D. (2016). Enhancing the feasibility of *Microcystis aeruginosa* as a feedstock for bioethanol production under the influence of various factors. Biomed. Res. Int..

[16] Fimbres-Olivarría D., López-Elías J.A., Carvajal-Millán E., Márquez-Escalante J.A., Martínez-Córdova L.R., Miranda-Baeza A., Enríquez-Ocaña F., Valdéz-Holguín J.E., Brown-Bojórquez F. (2016). *Navicula* sp. sulfated polysaccharide gels induced by Fe(III): Rheology and microstructure. Int. J. Mol. Sci..

[17] Mooij P.R., de Jongh L.D., van Loosdrecht M.C., Kleerebezem R. (2016). Influence of silicate on enrichment of highly productive microalgae from a mixed culture. J. Appl. Phycol..

[18] Markou G., Depraetere O., Vandamme D., Muylaert K. (2015). Cultivation of *Chlorella vulgaris* and *Arthrospira platensis* with recovered phosphorus from wastewater by means of zeolite sorption. Int. J. Mol. Sci..

[19] Bhatt N.C., Panwar A., Bisht T.S., Tamta S. (2014). Coupling of algal biofuel production with wastewater. Sci. World J..

[20] Patel D.S., He X., MacKerell A.D. (2015). Polarizable empirical force field for hexopyranose monosaccharides based on the classical Drude oscillator. J. Phys. Chem. B.

[21] Patel D.S., Pendrill R., Mallajosyula S.S., Widmalm G., MacKerell A.D. (2014). Conformational properties of α- or β-(1→6)-linked oligosaccharides: Hamiltonian replica exchange MD simulations and NMR experiments. J. Phys. Chem. B.

[22] Park D., Jagtap S., Nair S.K. (2014). Structure of a PL17 family alginate lyase demonstrates functional similarities among exotype depolymerases. J. Biol. Chem..

[23] Mühlroth A., Li K., Røkke G., Winge P., Olsen Y., Hohmann-Marriott M.F., Vadstein O., Bones A.M. (2013). Pathways of lipid metabolism in marine algae, co-expression network, bottlenecks and candidate genes for enhanced production of EPA and DHA in species of Chromista. Mar. Drugs.

[24] Dai L., Tan L., Jin X., Wu H., Houbo W., Tao L., Wenzhou X. (2020). Evaluating the potential of carbohydrate-rich microalga *Rhodosorus* sp. SCSIO-45730 as a feedstock for biofuel and β-glucans using strategies of phosphate optimization and low-cost harvest. J. Appl. Phycol..

[25] Blockx J., Verfaillie A., Thielemans W., Muylaert K. (2018). Unravelling the mechanism of chitosan-driven flocculation of microalgae in seawater as a function of pH. ACS Sustain. Chem. Eng..

[26] Chng L.M., Lee K.T., Chan D.J.C. (2017). Synergistic effect of pretreatment and fermentation process on carbohydrate-rich *Scenedesmus dimorphus* for bioethanol production. Energy Convers. Manag..

[27] Corrêa D.O., Duarte M.E.R., Noseda M.D. (2018). Biomass production and harvesting of *Desmodesmus subspicatus* cultivated in flat plate photobioreactor using chitosan as flocculant agent. J. Appl. Phycol..

[28] Gerchman Y., Vasker B., Tavasi M., Mishael Y., Kinel-Tahan Y., Yehoshua Y. (2017). Effective harvesting of microalgae: Comparison of different polymeric flocculants. Bioresour. Technol..

[29] Varshney P., Beardall J., Bhattacharya S., Wangikar P.P. (2018). Isolation and biochemical characterisation of two thermophilic green algal species—*Asterarcys quadricellulare* and *Chlorella sorokiniana*, which are tolerant to high levels of carbon dioxide and nitric oxide. Algal Res..

[30] Berteotti S., Ballottari M., Bassi R. (2016). Increased biomass productivity in green algae by tuning nonphotochemical quenching. Sci. Rep..

[31] Schulze P.S.C., Pereira H.G.C., Santos T.F.C., Schueler L., Guerra R., Barreira L.A., Perales J.A., Varela J.C.S. (2016). Effect of light quality supplied by light emitting diodes (LEDs) on growth and biochemical profiles of *Nannochloropsis oculat* and *Tetraselmis chuii*. Algal Res..

[32] Gupta S.K., Kumar N.M., Guldhe A., Ansari F.A., Rawat I., Nasr M., Bux F. (2018). Wastewater to biofuels: Comprehensive evaluation of various flocculants on biochemical composition and yield of microalgae. Ecol. Eng..

[33] Krayesky-Self S., Phung D., Schmidt W., Sauvage T., Butler L., Fredericq S. (2020). First report of endolithic members of *Rhodosorus marinus (Stylonematales, Rhodophyta)* growing inside rhodoliths offshore Louisiana, Northwestern Gulf of Mexico. Front. Mar. Sci..

[34] Li T., Xu J., Wu H., Jiang P., Chen Z., Xiang W. (2019). Growth and biochemical composition of *Porphyridium purpureum* SCS-02 under different nitrogen concentrations. Mar. Drugs.

[35] Dolganyuk V., Andreeva A., Budenkova E., Sukhikh S., Babich O., Ivanova S., Prosekov A., Ulrikh E. (2020). Study of morphological features and determination of the fatty acid composition of the microalgae lipid complex. Biomolecules.

[36] Ho S.-H., Huang S.-W., Chen C.-Y., Hasunuma T., Kondo A., Chang J.-S. (2013). Characterization and optimization of carbohydrate production from an indigenous microalga *Chlorella vulgaris* FSP-E. Bioresour. Techno..

[37] Chou N.T., Cheng C.F., Wu H.C., Lai C.P., Lin L.T., Pan I.H., Ko C.H. (2012). *Chlorella sorokiniana*-induced activation and maturation of human monocyte-derived dendritic cells through NF-κB and PI3K/MAPK pathways. Evid. Based Complement. Altern. Med..

[38] Liu Q., Yao C., Sun Y., Chen W., Tan H., Cao X., Xue S., Yin H. (2019). Production and structural characterization of a new type of polysaccharide from nitrogen-limited *Arthrospira platensis* cultivated in outdoor industrial-scale open raceway ponds. Biotechnol. Biofuels.

[39] El-Naggar N.E., Hussein M.H., Shaaban-Dessuuki S.A., Dalal S.R. (2020). Production, extraction and characterization of *Chlorella vulgaris* soluble polysaccharides and their applications in AgNPs biosynthesis and biostimulation of plant growth. Sci. Rep..

[40] Gaignard C., Laroche C., Pierre G., Dubessay P., Delattre C., Gardarin C., Gourvil P., Probert I., Dubuffet A., Michaud P. (2019). Screening of marine microalgae: Investigation of new exopolysaccharide producers. Algal Res..

[41] Dolganyuk V., Belova D., Babich O., Prosekov A., Ivanova S., Katserov D., Patyukov N., Sukhikh S. (2020). Microalgae: A promising source of valuable bioproducts. Biomolecules.

[42] Sero E.T., Siziba N., Bunhu T., Shoko R., Jonathan E. (2020). Biophotonics for improving algal photobioreactor performance: A review. Int. J. Energy Res..

[43] Toor M., Kumar S.S., Malyan S.K., Bishnoi N.R., Mathimani T., Rajendran K., Pugazhendhi A. (2019). An overview on bioethanol production from lignocellulosic feedstocks. Chemosphere.

[44] Yamin W.A., Shaleh S.R.M., Ching F.F., Othman R., Manjaji-Matsumoto M., Mustafa S., Shigeharu S., Kandasamy G. (2019). Harvesting Chaetoceros gracilis by flocculation using chitosan. IOP Conf. Ser. Earth Environ. Sci..

[45] Zhu Q., Wu S. (2019). Water-soluble beta-1,3-glucan prepared by degradation of curdlan with hydrogen peroxide. Food Chem..

[46] Rojo-Cebreros A.H., Ibarra-Castro L., Martínez-Brown J.M., Velasco-Blanco G., Martínez-Téllez M.A., Medina-Jasso M.A., Nieves-Soto M., Quintana-Zavala D., Jan M., Kazik P. (2017). Potential of Nannochloropsis in beta glucan production. Nannochloropsis: Biology, Biotechnological, Potential and Challenges.

[47] Welham S.J., Gezan S.A., Clark S.J., Mead A. (2014). Statistical Methods in Biology: Design and Analysis of Experiments and Regression.

